# Evaluation of resilience engineering using super decisions software

**DOI:** 10.15171/hpp.2019.27

**Published:** 2019-08-06

**Authors:** Gholam Abbas Shirali, Leila Nematpour

**Affiliations:** Department of Occupational Safety and Health Engineering, Faculty of Health, Ahvaz Jundishapur University of Medical Sciences, Ahvaz, Iran

**Keywords:** Analysis network process, Safety management systems, Resilience engineering, Super decisions software

## Abstract

**Background: ** Resilience engineering (RE) is a new approach to upgrade safety management systems. Due to its novelty in the field of safety, RE seems to be promising in providing good indicators to assess priorities in organizational strengths/weaknesses while planning to promote safety within organizations. Several methods have been recently developed to evaluate REperformance. The current study is an attempt to quantify and determine the priorities of REdimensions in a steel industry using analytic network process (ANP).

**Methods: ** In this cross-sectional study, 489 male workers of a steel industry participated. For this purpose, the RE questionnaire was distributed among workers and, then, super decisions software (version 3.2) was used to analyze the data.

**Results: ** The results indicated that there was a sufficient level of RE in the organization where top management commitment with normalized weight 0.1781 and awareness-opacity with normalized weight 0.1483 were ranked as the first and last priorities of the organization, respectively.

**Conclusion:** The results of this study showed that the top management system, with the adoption of safety policies, has been able to improve the performance of RE in the organization. Managers should consider appropriate measures to improve the RE situation.

## Introduction

One of the best strategies for improving safety performance is the proactive approach to safety management. An effective safety management can contribute to the reduction of occupational accidents by helping managers create safe working environments for workers.^1,2^ Today, various studies have reported organizational factors, functional variability and, the combination of a series of unpredictable events as the main causes of accidents, especially in social-technical systems.^3,4^ Currently, resilience engineering (RE) has provided an important arena for the comprehension of safety management in social-technical systems,^5^ has brought about some changes in attitudes towards safety maintenance, and has enhanced the safety of complex systems. These changes have led to the introduction of a new approach for analyzing the positive role of individuals at all organizational levels rather than laying emphasis on human error.^6^ Resilience refers to an organization’s capability to sustain or rapidly return to a steady state that allows the organization to continue operation during or after a major event or in the presence of continuous stresses.^7^ RE helps people succeed in focusing on the complexities that have put them under pressure. Emphasizing the characteristics of RE, people learn how to provide safe conditions to adapt themselves to changes and surprises, dangers as well as diverse and inconsistent goals.^8^ RE analytical framework consists of individual, team, and organizational levels.^9^ At each of these levels, resilience includes three dimensions: 1) The ability to prevent incident; 2) The ability to prevent the spread of incident consequences, and 3) The ability to recover to normal conditions after an incident.^9^ Hollnagel and Woods have proposed six indicators for assessing RE potentials where it becomes possible to identify the potential concerns and functionality of systems.^9^ These six principles are as follows:

- *Top Management Commitment:* The commitment of top managers to implement the safety and health system towards the fulfilment of organizational goals is the most important objective of any organization.^7^
- *Just culture:* It refers to the creation of a safe and trustworthy environment for employees to report important safety issues.^10^
- *Culture of learning:* It means that individuals must try to learn not only from incidents and events in emergency situations to prevent repetition of mistakes, but to learn work concepts in normal and standard conditions.^11^
- *Awareness-opacity:* This means that employees should be aware of the current state and defending state of the organization as well as the boundaries of the system. They should also be mindful of potential risks to assess safety and development.^11,12^
- *Preparedness:* The system is always attempting to have the foresight to provide solutions for various threats.^12^
- *Flexibility:* The system should adapt itself to complex and difficult conditions, recover from critical conditions, and prevent failures.^12^

Analysis network process (ANP) is a systematic approach for selecting alternatives and making decisions about problems where the concepts of fuzzy theory and analytic network process are used. An open source of ANP architecture allows for modeling different selection criteria regardless of the indicators’ priority.^13^ In this approach, the complex relationships between decision elements are taken into account by the network structure and each subject is considered as a network of criteria, sub-criteria, and alternatives that have come together in a cluster. All elements in a network can be interrelated in some way. In other words, feedback and interaction among clusters are possible in networks.^14^ The ANP approach has been developed to compensate for the weaknesses of analytic hierarchical process (AHP) approach wherein the necessary computations are performed through simpler multi-criteria decision-making. However, many of the structured decision-making problems are not of a hierarchical nature, and this can be attributed to the complexity and dynamic nature of the problems. Therefore, the interaction among the elements of the upper levels and those of the lower levels and the interaction among the elements of a given level are allowed in the ANP approach.^15^ Where dependencies are two-sided, the problem exits from the hierarchical mode and forms a nonlinear network or system where the rules and formulas of AHP approach are not applicable anymore. In these cases, the elements should be weighted by the ANP approach proposed by Saati in 1994.^16^ The most important weighting method in ANP is based on the eigenvector method, which is calculated by super decisions software. The dependency structure matrix can be based on the relationships between criteria and even the relationship between a criterion and a sub-criterion. Dependencies may be bilateral or unilateral. In this case, the control or target criterion can be considered as one of the criteria. In the analytic network analysis, the final weighting is determined by super-matrices that are composed of the dependency and non-dependency matrices of the criteria and sub-criteria, which lie in their right place; and the final weight is calculated through mathematical operations.^16^

Li et al studied a preventive process of risk evaluation approach based on the analysis of occupational risk and RE. In their research, the risks of each stage of gas transfer process and resilience risk reduction actions were evaluated by the combination of JHA method and RE theory compared with HAZOP method. The results indicated that JHA was not sufficient for evaluating the preventive risk because it is systematically not appropriate enough for covering all the probable actions, including preventive and improving actions.^17^ In a study of this kind, Said et al proposed a new structure of technological social systems by means of RE. In this study, they used a system modeling method on resilience process behavior in a critical point, called causal loop diagram for the qualitative evaluation of variables and system communications. Finally, they created a scoring system for evaluating and rating the resilience capacity based on the results.^18^ In a study carried out by Hashemi et al, an adaptive algorithm was proposed for evaluating the performance of construction project management based on resilience management and job security. The proposed algorithm included radial basis function, artificial neural networks multi-layer perceptron, and statistical tests. The results showed that readiness and flexibility were the factors affecting the total efficiency. In addition, resilience management and job security exerted similar effects on the total efficiency of the system.^19^ In another study, Omidvar et al presented a model for assessing the performance of an organization based on RE using a fuzzy AHP in the petrochemical industry. They presented management commitment and preparedness in the face of emergency conditions and used these main two factors to determine the resilience level. These factors could make the highest contribution to maintaining the organization status within acceptable limits.^20^ Shirali et al evaluated RE factors based on system properties in a process industry by principal component analysis and numerical taxonomy. The results showed that resilience had the determination power of poor indicators and units in the industry.^21^

The comparison of this study with other studies shows that the present study is the only one that has examined the performance of RE in a steel industry using ANP method. The literature review shows that a large number of studies have been conducted in the field of RE. In the present study, it is sought to assess safety performance in a steel company based on RE and to prioritize its indicators from workers’ viewpoint by using ANP approach. It is possible to propose solutions to improve the safety status and reduce accidents through the quantitative evaluation of weak aspects of resilience and its function in the industry. As a result, it is possible to evaluate the organizational resilience in critical situations and its potential to recover to a sustainable model. Therefore, line employees were the target of this research because they are in the front-line of production.

## Materials and Methods

### Research strategies and sample

The case plant was a large steel industry in Iran with more than 1000 employees. The employees worked in three shifts, each of which took 8 hours. They were required to perform their tasks in a context with a high degree of function complexity, work demand, and production pressure. A validated RE questionnaire was used for data collection.^4^ This questionnaire consists of six indicators, namely top management commitment, just culture, learning culture, awareness and opacity, flexibility, and preparedness; and included 61 items (see Supplementary file 1). The responses were scored based on a 5-point Likert scale (from “strongly disagree” to “strongly agree”). The scores were calculated based on the number of questions in the field and the responses provided by employees.^22^ The case plant was a steel industry and had been classified into six units, namely central workshop, crane unit, department of engineering, electrical automation unit, orders unit, production unit, and stockroom. The sample size was estimated equal to 546 with respect to the pre-test (error: 0.05).

Accordingly, the questionnaire was distributed among those employees who were more likely to be affected by occupational risks. Validity and reliability of questionnaire was previously conducted by Shirali et al.^4^ The respondents were assured that their responses would remain confidential and would be used solely for research purposes. A total of 489 valid questionnaires were gathered from a 546-participant population. Thus, the response rate of the study was 89.56%. Finally, the data obtained from the RE questionnaire completed by the employees were inserted into Super Decisions software (version 3.2) and were analyzed in the direct section. Then, the priority of each indicator was obtained and the necessary suggestions were made to improve the resilience condition.

### Execution of ANP Model

**Figure 1:**
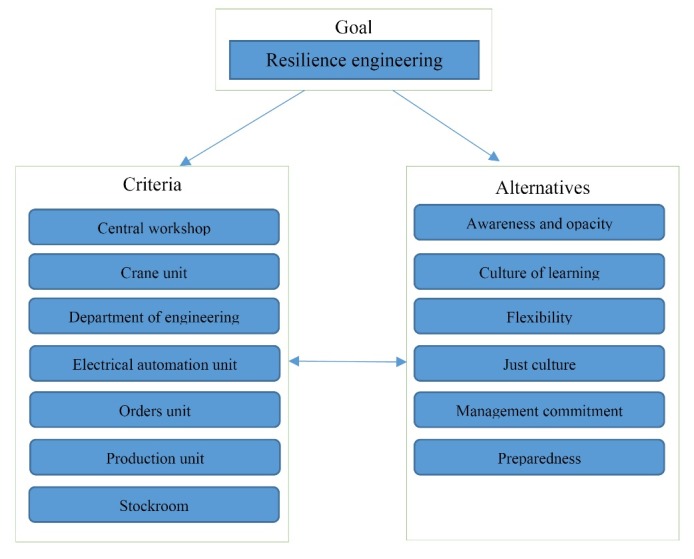
Resilience Engineering ANP.

In order to execute the ANP model, it is necessary to form a suitable network model that encompasses the research objective as well as the identified main components, criteria, and sub-criteria to cover the research objectives. Figure 1 presents the network model, which is used to evaluate RE indicators in the selected units of the steel industry. One way of performing calculations in ANP method is to put the weights obtained from pairwise comparisons in a matrix, called super matrix. It is a matrix of the relationship between the network components and is obtained from the eigenvectors of these relationships. Super matrix can be divided into different blocks where each block indicates the weight obtained from the pairwise comparison of the rows (for example, indicators) with regard to the columns (for example, the choices or indicators).^16^ To run the ANP, it is necessary to specify the interactions among the components, while there may be no interaction among them. However, such interactions, if existing, may be unilateral or bilateral. In this method, the inconsistency ratio is equal to 0.512, which is smaller than 0.100.

### Calculation of limited super matrix 

An unweighted super matrix is created from the addition of the internal priority vectors pertaining to the importance coefficients of the components and clusters in the elementary super matrix. Subsequently, the weighted super matrix is calculated from the multiplication of unweighted super matrix by the cluster matrix. After normalizing the weighted super matrix, the super matrix turns into a random mode in terms of columns. In the end, the limit super matrix is calculated by the exponentiation of all elements of the weighted super matrix. An ANP-based prioritization presents a cluster in a normalized, limited, and graphical network model. Super matrices are calculated in three modes. The first mode is unweighted and includes the relative priorities obtained from the pairwise comparison within the network. The second weighted super matrix is obtained through multiplying all the elements of the unweighted super matrix by the corresponding elements of clusters’ weights. The third super matrix (limit) is obtained by consecutively raising the weighted super matrix to power. When the numbers are the same for each column, the limit matrix has been obtained and the matrix multiplication process is halted. Finally, the weights obtained from the prioritization of the RE indicators are normalized in order to normalize the research results.^16^

## Results

In this study, in the first stage, the level of RE at each individual unit was determined by the software and the results are presented in Table 1. As the results indicate, the first column is graphical where the normal column displays the priority of each alternative based on the paired comparison form and this is the most common method for the observation of the results. The ideal column indicates the obtained results by dividing the values in either the normalized or the limiting columns by the largest value in the column. Therefore, the value of the selected alternative always equals 1.00. The values of total or raw columns are obtained directly from the limit super matrix.^16^

The three levels of RE have been prioritized in Table 1. The results show that the electricity automation unit and the order unit enjoy the highest and lowest levels of RE in the steel industry, respectively.

**Table 1:** Prioritization of resilience culture in the Steel Industry for each unit

**Alternatives**	**Total**	**Normal**	**Ideal**	**Ranking**	**Graphic**
Central workshop	0.0687	0.1375	0.9225	6	0.9225
Crane unit	0.0732	0.1464	0.9825	3	0.9825
Department of engineering	0.0696	0.1392	0.9337	5	0.9337
Electrical automation unit	0.0745	0.1490	1.0000	1	1
Orders unit	0.0679	0.1358	0.9110	7	0.911
Production unit	0.0740	0.1480	0.9931	2	0.9931
Stockroom	0.0721	0.1441	0.9669	4	0.9669

In the next stage, the level of RE was analyzed by each individual resilience indicator in the steel industry and the results have been presented in the prioritized format in Table 2. According to the findings of this study, it was found that management commitment had the highest rank while awareness-opacity had the lowest rank in the industry.

**Table 2:** Prioritization of resilience Culture in the Steel Industry for each resilience index separately

**Alternatives**	**Total**	**Normal**	**Ideal**	**Ranking**	**Graphic**
Awareness and opacity	0.0742	0.1483	0.8326	6	0.8326
Culture of learning	0.0871	0.1742	0.9778	3	0.9778
Flexibility	0.0829	0.1657	0.9302	4	0.9302
Just culture	0.0880	0.1760	0.9880	2	0.988
Management commitment	0.0891	0.1781	1.0000	1	1
Preparedness	0.0788	0.1576	0.8847	5	0.8847

**Figure 2:**
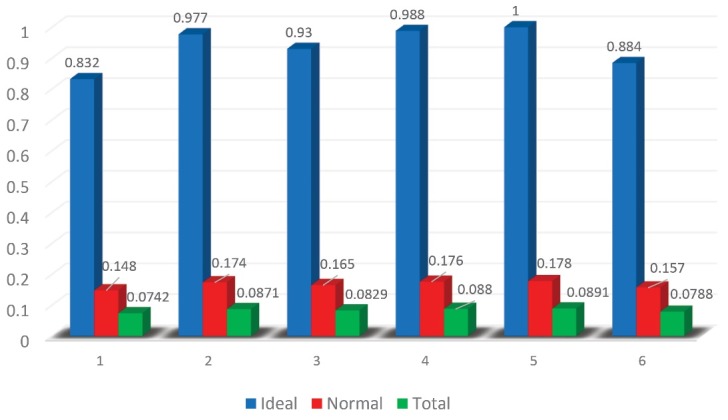
Final priorities of ANP: 1. Awareness and Opacity 2. Culture of Learning 3. Flexibility 4. Just Culture 5. Management Commitment 6. Preparedness.

Figure 2 shows individual priorities and their distance from each other, as presented in Table 2.

Figure 2 shows, indicators of the resilience were proximity together. The ideal weight of management commitment 1 and awareness and opacity 0.832 have been the highest and lowest, respectively. Also, the normalized weight of management commitment was 0.178 and awareness and opacity was 0.148.

## Discussion

In this study, it was attempted to prioritize different levels of resilience using ANP technique in different units of a steel industry. As in Table 2, RE is of the highest rank in the electricity automation unit, which represents the highest level of resilience in this unit, whereas the production unit lies in the second priority. These results show that the higher ranking of resilience in the electricity automation unit may be attributed to the fact that this unit is usually run by educated operators and that these operators are more concerned with safety. Research findings about on safety culture show that there is a significant correlation between safety culture and operators’ level of education.^23,24^ This can be attributed to the effect of education level on operators’ attitudes towards safety. In fact, the operators with higher education have more positive attitudes toward safety and, consequently, show a low interest in unsafe actions. Since educated individuals have a higher awareness of safety issues, have a better understanding of safety teachings, and comprehend safety instructions more effectively; they try to adhere to them at workplace.^25,26^ The production unit was ranked second because of its high significance for the organization, sustainable trainings, and its greater focus on safety and management. This can increase the RE level and reduce the risk of accidents in the industry. To interpret the findings of this study regarding the level of resilience and the education level in the production unit, it can be argued that the training courses held for individuals with different levels of education in the steel industry have made all employees’ level almost equal because operators with different levels of education take advantage of safety training and health teaching equally. Therefore, according to the results, it can be concluded that the promotion of safety and resilience culture requires special planning.^27^ Shirali et al performed a quantitative evaluation of resilient safety culture by analyzing the main components and numerical classifications in a petrochemical industry. The results indicated that different components of resilient safety culture have lower scores compared with other components.^28^ The ordering unit enjoyed the lowest level of resilience in the steel industry, and this is indicative of the assignment of inadequate attention this unit on the part of executors.

The RE indicators of the steel company have been prioritized in Table 2. The results presented in this table show that top management commitment has been ranked first in terms of workers’ attitudes towards the respective units. This reflects the considerable focus of top managers on the unit under their control. This finding of the present study is consistent with those of resilience studies conducted in other industries.^20,29,30^ In a study, Jafari et al evaluated resilience indicators in critical systems of a refinery. According to the experts, senior management commitment is one of the organizational factors that affect resilience and is the main factor of success for safety plans.^31^ The just culture has been ranked second and this represents that top managers have provided a safe environment for workers so that they can easily report incidents and mistakes to the authorities. Pinion et al pointed out that top management commitment to safety increases self-reporting among workers.^32^ The last priority from the workers’ viewpoint has been assigned to awareness-opacity; that is, the workers have the least awareness of the current situation and the defensive state of the organization, and do not either have enough knowledge about the system limits. Workers’ awareness of possible risks is very low and they do not have the ability to assess safety and production. From the workers’ perspective, preparedness is the fifth priority of this organization. Preparedness means the prediction of unwanted events, as well as the ability to react appropriately when an unforeseen incident occurs.^33^ In other words, when an organization acts weakly in the face of problems and does not have the ability to predict potential problems, it lacks preparedness. This means that no arrangements and measures have been taken into account for identifying and assessing risks, and no plans have been made for reacting to emergencies, and the necessary trainings have not been designed. This will prevent workers from participating in safety actions. Various studies have considered preparedness as one of the indicators with the greatest impact on resilience level.^32,34-37^ Learning culture and flexibility have been ranked third and fourth, respectively. One of the most important actions of managers and supervisors, indicating their practical support for safety, is the provision of safety training.^38^ Studies show that the learning culture has the greatest impact on management and organizational factors, and this is representative of the effective role of training in the safety culture of an organization.^30,39,40^ As for resilience, organizations must be able to adapt to new and complex problems, and this calls for the capability to make critical decisions without having to wait for management guidelines at workplace.^41^ In this domain, Zarrin and Azadeh investigated the effects of RE on health, safety, environment, and ergonomic management system by using Z-number cognitive map. The results showed that senior management commitment (0.827) had the highest effect on the environment, learning (0.792) had the highest effect on health, readiness (0.786) had the highest effect on ergonomics, and awareness (0.776) had the highest effect on safety.^42^

One of the limitations of the study was evaluation of the importance of indicators based on the workers’ opinions. Whereas, by using objective methods (such as Shannon entropy), effectiveness and efficiency of abstract methods can be increased. Therefore, it is suggested to use abstract-objective combined evaluation method for evaluating resilience indicators. Also, increase the population of study and use of the occupational health and safety experts’ opinions can help to better resilience prioritization in steel industries.

## Conclusion

According to the current findings, top management commitment was revealed as the most effective indicator and, thereby, it can be used to determine the level of resilience. The top managers of an organization are the creators of culture in that organization. In this way, they can contribute to the creation and sustainability of cultures and, consequently, improve organizational resilience by means of financial planning, active participation, auditing, inspection, and other factors. When managers realize that a crisis has happened, they usually plan for devising a crisis settlement strategy in accordance with standardized guidelines. Nonetheless, when a non-precedence crisis occurs, there is no standardized guideline to deal with that. Therefore, operation instructions or crisis management plans should be so flexible and transparent that they can accommodate unpredictable variables^43^. In addition, awareness, preparedness, and opacity were revealed to lie among the weak points of resilience in the organization. As a result, managers should take appropriate measures to improve this situation.

## Ethical approval

Not Applicable.

## Competing interests

The authors declare that they have no competing interests.

## Funding

There is no funding resource.

## Authors’ contributions

These authors contributed equally to this work.

## Acknowledgments

The present article is a part of one practical project on safety which was conducted with the spiritual support of the Steel Company. The authors would like to express their thankfulness to the safety manager and employees of the Steel Company for their contribution to this study.

## Supplementary Materials


Supplementary file 1 contains Resilience engineering survey questionnaire.
