# Supplementary material for: Evaluation of resilience engineering using super decisions software
Source: Health Promot Perspect. 2019 Aug 6;9(3):191–7. doi: 10.15171/hpp.2019.27 (PMC6717918; doi:10.15171/hpp.2019.27)
Supplement: Supplementary file 1 — contains Resilience engineering survey questionnaire. [file hpp-9-191-s001.pdf]

Supplementary file 1. Resilience engineering survey questionnaire

Management commitment

1. My superior director appreciates my work.
2. The appreciation which the superior director shows for my work is completely visible.
3. In my opinion, the appreciation which the superior director shows staff under his or her.
4. Production is always the first and most valuable priority and of my superior manager.

Awareness and opacity

5. When the safety/resilience is in danger, I can stop production and staff should be encouraged to do so.
6. After unfortunate events, the first priority of management is to find and correct faulty barriers or safeguards, not to search for scapegoats to blame.
7. My superior manager provides enough resources and facilities to keep up and upgrade safety/resilience.
8. My superior manager is always aware of organizational, human and technological risks, which can threaten the organization's activities.
9. My superior manager gives me constructive feedbacks regarding work and safety/resilience.

Just culture

10. If staffs have concerns about their safety/resilience and work, they can consult with their superiors.
11. Reporting problems and safety/resilience issues to the superior is easy.
12. My superior director tolerates any news especially bad news.

13. Staffs who report a problem of safety/resilience or offer a mechanism to improve safety are encouraged.
14. In my workplace, staffs can affect the decisions of their superiors.
15. If staffs have concerns about their safety/resilience of work, they must not do it.
16. Decision making on issues related to safety/resilience in a system in which I'm working is a partnership.
17. In a system in which I'm working, staffs of different departments and levels take part in safety/resilience meetings.
18. In a system in which I'm working, the spirit of team work completely “is tangible”.
19. In a system in which I'm working, safety/resilience function is a part of the staff evaluation system.
20. Administrative bureaucracy (paperwork), impacts my workplace safety/resilience.

Culture of learning

21. If the system under my management fails, I am aware of the consequences.
22. Information about the flaws and shortcomings of the system must be reported to the competent people of the organization.
23. Defects must be carefully analyzed and the reasons for their occurrence must be explained to the other staff to learn a lesson from them and to prevent a repeat.
24. Discussion and an exchange of views about the risk in my organization or workplace are very important to me.
25. Results of feedbacks or revisions made during occurrence of the events in the organization are used for the corrective actions.
26. Can you be sure that the event that did not happen in the past will not happen in the future?

27. I think that my personnel strive actively to create a good culture of learning.
28. Training courses in my organization or workplace are proper for promoting safe behaviors.
29. In my organization or workplace, retraining courses are conducted regularly and at the appointed time.
30. I have received necessary training to do the job properly and safely.
31. I can devise a way to learn a lesson from the present defects in my workplace.
32. Instructions and manuals are old and are not suitable for performing work safely.
33. Manuals have only an “aspect of the guidance” and their use is not mandatory.
34. Instructions and safety/resilience rules at the workplace are regularly updated.
35. The person responsible for safety/resilience is clearly defined for each operation.

Awareness and opacity

36. The organization expects that I do several tasks simultaneously.
37. Most of these tasks have conflicting goals.
38. I have to do my job even with little information about it.
39. If a conflict exists between safety/resilience and production, safety is the priority.
40. I usually have enough time to do my job.
41. The safety/resilience quality drastically influences the financial and budgetary issues.
42. The staffs understand and conduct entire training related to the safety/resilience.
43. Result of the safety/resilience trained by staff can also be seen in action.
44. There are good safe ways to do my job that I'm aware of.
45. In the emergency condition and rapid response, to follow instructions is easy.
46. I am aware of major worries and concerns about safety/resilience in the organization or my workplace.

Preparedness

47. Resilience safety culture and rules governing my organization and workplace are appropriate and they can also be used in the future.
48. Holding group meetings or workshops in the areas of safety/resilience is the perfect solution to expect potential problems in the future.
49. I prefer to talk about my concerns regarding safety/resilience with my coworkers and superiors.
50. Encouraging staffs that are able to find future events or risks (a sixth sense) is essential.
51. Part of a limited budget of safety/resilience should be spent on improving safety or resilience and preparedness to deal with future problems.
52. Often, unexpected events occur at my workplace.
53. At my workplace, incidents possibility is high.
54. In my workplace, safety/resilience issues steadily are raised and surveyed at the level of senior management and not as a single case, or only after the adverse events.
55. We need comprehensive and complete information to plan in order to deal with possible accidents that may come in the future.

Flexibility

56. My access to the helping resources (facilities, time, etc.) is ideal for dealing with unexpected events.
57. If I am faced with a problem such that I have to sacrifice safety/resilience or production, I prefer to select safety/resilience for keeping the system.
58. In a major emergency and critical condition for important decision making (such as stop production) permission from my superiors is necessary.

59. The organization where I work has the necessary facilities and procedures to respond to unpredictable and unexpected changes and disruptions.
60. The organization where I work has the ability to adapt to stressful situations caused by internal and external pressures.
61. If the system collapses, it has the ability to restructure and return quickly to its original state (stable).
